# A Review on Vital Pulp Therapy in Primary Teeth

**Published:** 2014-12-24

**Authors:** Iman Parisay, Jamileh Ghoddusi, Maryam Forghani

**Affiliations:** a * Dental Materials Research Center and Department of Pediatric Dentistry, Dental School, Mashhad University of Medical Sciences, Mashhad, Iran; *; b * Dental Research Center and Department of Endodontics, Dental School, Mashhad University of Medical Sciences, Mashhad, Iran*

**Keywords:** Calcium-Enriched Mixture, Mineral Trioxide Aggregate, Primary Teeth, Pulp Capping, Pulpotomy, Vital Pulp Therapy

## Abstract

Maintaining deciduous teeth in function until their natural exfoliation is absolutely necessary. Vital pulp therapy (VPT) is a way of saving deciduous teeth. The most important factors in success of VPT are the early diagnosis of pulp and periradicular status, preservation of the pulp vitality and proper vascularization of the pulp. Development of new biomaterials with suitable biocompatibility and seal has changed the attitudes towards preserving the reversible pulp in cariously exposed teeth. Before exposure and irreversible involvement of the pulp, indirect pulp capping (IPC) is the treatment of choice, but after the spread of inflammation within the pulp chamber and establishment of irreversible pulpitis, removal of inflamed pulp tissue is recommended. In this review, new concepts in preservation of the healthy pulp tissue in deciduous teeth and induction of the reparative dentin formation with new biomaterials instead of devitalization and the consequent destruction of vital tissues are discussed.

## Introduction

Premature loss of primary teeth can lead to malocclusion besides functional and esthetic problems. Therefore, preserving the vitality of deciduous teeth until their natural exfoliation time is critical for maintaining the arch integrity. The pulp in primary dentition is histologically similar to permanent teeth and may be affected by caries, restorative procedure and trauma. Depending on severity of injury; the reaction of pulp is different [1]. However, this similarity is not applicable when pulp reaction to irritants is concerned [2]. Accepted endodontic therapy for primary teeth can be divided into two main categories: vital pulp therapy (VPT) and root canal treatment (RCT). The primary objectives of VPT in deciduous teeth are treating reversible pulpal injuries and maintaining pulp vitality/function. Several factors such as adequate blood supply, severity of inflammation, obtaining homeostasis, disinfection of the exposure site, antibacterial properties and biocompatibility of pulp covering agents and adequate coronal seal may affect the success of VPT. The most important factor in success of VPT is vitality of the pulp and, in particular, the presence of proper vascularization, which is necessary for active formation/function of the odontoblasts.

VPT includes three therapeutic approaches: indirect pulp capping (IDPC) for teeth with dentinal cavities and reversible pulpitis; direct pulp capping (DPC) and pulpotomy [1] which are considered in cases of pulp exposure. This review intended to provide information on different approaches of VPT for primary teeth.

***Indirect pulp capping (IDPC)***

IDPC is recommended for teeth with deep carious lesions approximating the pulp but there are no signs or symptoms of pulp degeneration. In this procedure, the deepest layer of the remaining carious dentine is covered with biocompatible materials [1].

Several medicaments are advocated for IDPC such as mineral trioxide aggregate (MTA) [3], medical Portland cement (PC) [3], calcium hydroxide (CH) [4], resin modified glass ionomer (RMGI) [5, 6], dentin bonding agents [4] and bioactive molecules such as enamel matrix protein (Emdogain) or members of bone morphogenic protein (BMP) super family such as tissue growth factor-β (TGF-β) [1].

The rationale for IDPC is that few viable bacteria remain in the deeper dentine layers and after the cavity has been seated properly, they will be inactivated. Based on the clinical studies that looked precisely at the partial caries removal and residual bacteria, there was a dramatic reduction in the colony forming units (CFU) of bacteria regardless of using either zinc oxide-eugenol (ZOE) or CH on the remaining carious lesion. This result focuses on the importance of cavity seal and may negate the need for re-entry in these cases unless symptoms prevail [7].

Success rate of IDPC have been reported to be higher than 90% in primary teeth [8, 9]. Several studies reported the success rate of IDPC with different agents in primary teeth which are summarized in Table 1.

According to the growing evidence about the success rate of IDPC in deciduous teeth, this treatment approach can be recommend as an appropriate strategy for symptom-free primary teeth with deep carious lesions provided that a proper leakage-free restoration can be placed.

***Direct pulp capping (DPC)***

Direct pulp capping (DPC) is carried out when a healthy pulp has been mechanically/accidentally exposed during operative procedures or trauma. The injured tooth must be asymptomatic and the exposure site must be pinpoint in diameter and free of oral contaminants [1]. DPC involves the application a bioactive dental material on the exposed pulp in an attempt to preserve its vitality [10]. The rationale behind this treatment is to stimulate the pulp to initiate reparative tertiary dentine formation at the exposure site [11].

DPC of primary teeth is one of the most controversial treatment methods. The success rate of this method is not particularly high for deciduous teeth [1]. The undifferentiated mesenchymal cells which may differentiate into odontoclasts leading to internal resorption, are responsible for high failure rate of this treatment [1].

Several medicaments have been introduced for DPC including: CH [12, 13], ZOE cement [14], formocresol (FC) [12], polycarboxylate cement [14], dentine adhesives, enamel matrix derivative (EMD) [13], MTA [11, 15, 16], calcium-enriched mixture (CEM) cement [14] and simvastatin [17]. Some of the medicaments which present better results in trails are indicated in Table 2.

Although guidelines published by the American Academy of Pediatric Dentistry (AAPD) do not recommend DPC for caries exposed primary teeth [18], promising results (over 90% success) of recent clinical trials [11, 14, 17] may challenge this policy in the future.

***Pulpotomy***

Pulpotomy is one of the most widely accepted clinical procedures for treating cariously exposed pulps in symptom-free primary teeth. The rationale is based on the healing ability of the radicular pulp tissue following surgical amputation of the affected or infected coronal pulp [1].

Pulpotomy can be performed using different techniques including non-pharmacotherapeutic treatments such as electrosurgery (ES) [19] and laser [20-22] or pharmacotherapeutic approaches by dressing the pulp tissue with different medicaments or biological materials such as FC [23, 24], gultaraldehyde (GA) [25], ferric sulfate (FS) [26, 27], CH [28, 29], MTA [30, 31], freeze-dried bone [32], bone morphogenic protein (BMP) [33], osteogenic protein [34], sodium hypochlorite (NaOCl) [35, 36], CEM cement [37], enriched collagen solutions [38], PC [39] and fully synthetic nanocrystalline hydroxyapatite paste [40]. Pulpotomy also can be classified according to the following treatment objectives: devitalization (mummification, cauterization), preservation (minimal devitalization, noninductive) or regeneration (inductive, reparative) [41].

**Table 1:** Variable success rates with different indirect pulp capping (IDPC) agents (CHX=chlorhexidine, RMGI=resin modified glass ionomer, PC=Portland cement, CH=calcium hydroxide, GI=glass ionomer)

**Author, year**	**IDPC medicament**	**Success (%)**	**Follow-up (month)**	**Sample size (n)**	
				**Baseline**	**Final**
**Rosenberg ** ***et al.*** ** (** **2013) ** **[** 42 **]**	CHX and RMGI	97	12	60	32
**Petrou ** ***et al.*** ** (** **2013) ** **[** 3 **]**	MTA	90.3	6.3	11	9
	PC	90.3		13	9
	CH	90.3		14	10
**Trairatvorakul and ** **Sastararuji** ** (** **2013) ** **[** 43 **]**	CH	94	12-29	41	35
	3 MIX antibiotic	78		41	37
**Arizos** ** and Kotsanos (** **2011) ** **[** 5 **]**	RMGI	96.5	31	90	86
**Gruythuysen ** ***et al.*** ** (** **2010) ** **[** 6 **]**	RMGI	96	36	125	86
**Casagrande ** ***et al.*** ** (** **2009) ** **[** 44 **]**	Total etch adhesive	93	60	25	15
	CH	80	60	23	10
**Franzon ** ***et al.*** ** (** **2007) ** **[** 45 **]**	CH	73.3	36	19	15
	Gutta-percha	85.7			
**Marchi ** ***et al.*** ** (** **2006) ** **[** 46 **]**	CH	88.3	48	12	12
	RMGI	93.3		15	15
**Vij ** ***et al.*** ** (** **2004) ** **[** 8 **]**	GI	94	40	108	108
**Al-Zayer** *** et al.*** ** (** **2003) ** **[** 9 **]**	CH	95	14	187	

Although a considerable number of clinical trials with different techniques and materials have been performed and published about pulpotomy in primary teeth, a Cochrane review found that evidence is lacking to conclude which is the most appropriate technique for pulpotomies in primary teeth [47].

Among those biological materials and medicaments which were mentioned earlier, some of them are widely accepted and showed good clinical and radiographic success rates which will be discussed considering the treatment-objective classification.

***Devitalization***

The first approach in pulpotomy of deciduous teeth is devitalization, where the vital pulp tissue is destroyed. It includes pulpotomy with FC, GA, ES and laser.

***Formocresol: ***FC has been a popular pulpotomy medicament in the deciduous teeth for the last 70 years, since its introduction by Sweet in 1932 [48]. The success rate of FC pulpotomy is reported to be 70-98%. FC consists of 19% formaldehyde, 35% cresol in a vehicle of 15% glycerin and water (Buckley's solution) [36]. FC prevents tissue autolysis by bonding to protein; Berger [49] described the histological view of pulp tissue following FC pulpotomy. Fixation of the pulp occurred in coronal third of the root, the middle third presented loss of cellular integrity and apical third showed granulation tissue growth. Although concerns have been raised about safety (*i.e.* mutagenicity, carcinogenicity and immune sensitization potential) of FC application in human [37], no correlation between FC pulpotomies and cancer has ever been demonstrated [38].

***Gultaraldehyde: ***GA was introduced to dentistry in 1979 by Kopel [41]. It has been suggested as an alternative to FC as a pulpotomy agent based on its superior fixative properties, low antigenicity and low toxicity. GA causes rapid surface fixation of the underlying pulpal tissue. A narrow zone of eosinophillic, stain and compressed fixed tissue is found directly beneath the site of application, which blends into vital normal appearing tissue apically [41]. 

In a recent study by Havale *et al.* [50] the relative clinical and radiographic success of FC, GA and FS pulpotomies were 

compared at three-month intervals over one year. The clinical success rates of FC, FS and GA were 86.7, 96.7 and 100%, respectively. The radiographic success rates gradually decreased over the year in all pulpotomy groups and the radiographic success rates in FC, GA and FS were 56.7, 83.3 and 63.3%, respectively. Therefore, 2% GA may be recommended as an alternative to FC pulpotomy.

In the other clinical study by Tsai *et al.* [51], the clinical and radiographic success rates of 5% buffered GA were 98 and 87.5%, respectively. But the relative high failure rate in this long-term follow-up indicated that clinicians should be cautions before extensively using GA as a pulpotomy agent.

***Electrosurgery: ***ES is a non-pharmacological homeostatic technique which has been suggested for the pulpotomy procedure. It involves cutting and coagulating soft tissues by means of high-frequency electric current passing through the tissue cells [52]. This technique carbonizes and heat denatures the pulp and bacterial contamination. ES pulpotomy seems to have great merits. The self-limiting pulpal penetration is only a few cell layers deep. There is good visualization and homeostasis without chemical coagulation or systemic involvement. Spending less chair time in this technique than the FC pulpotomy is another benefit [27].

In a randomized clinical trial by Bahrololoomi *et al.* [53] no significant difference between ES and FC pulpotomies in primary molars were reported [53]. In another randomized clinical trial Dean *et al.* [19] found that there was not any significant difference between the success rates for ES and FC pulpotomy techniques which is similar to the results of a study by Rivera *et al.* [54]. 

In a randomized clinical trial, Farrokh Gisoure [27] compared the clinical and radiographic success rate of ES, FC, and FS pulpotomies of primary molars. The overall success rates of ES, FC and FS were 83.3, 82.1 and 87.5%, respectively. Favorable clinical and radiographic success rates of ES and FS pulpotomy was observed which was comparable to FC. Because of few clinical trials comparing ES to other pulpotomy techniques, further clinical studies must be conducted to reveal reliable results toward effectiveness of ES pulpotomy in primary teeth. 

**Table 2:** Variable success rates with different direct pulp capping (DPC) agents (FC=formocresol, EMD=enamel matrix derivatives, CH=calcium hydroxide)

**Author, year**	**DPC medicament**	**Clinical success (%)**	**Follow-up (month)**	**Sample size (n)**	
				**Baseline**	**Final**
**Fallahinejad ** ***et al.*** ** (** **2013) ** **[** 55 **]**	MTA	95	20	42	38
	CEM	89			
**Fallahinejad ** ***et al.*** ** (** **2010) ** **[** 14 **]**	MTA	100	6	21	19
	CEM	94.8	6	21	19
**Aminabadi ** ***et al.*** ** (2010) [** 12 **]**	FC	90	24	60	-
	CH	61.7	24	60	-
**Garrocho-Rangel ** ***et al.*** ** (** **2009)** ** [** 13 **]**	EMD	97	12	45	45
	CH	97	12	45	45
**Tuna and ** **Ölmez** ** (2008) [** 11 **]**	MTA	100	12	25	22
	CH	100	12	25	22
**Caicedo** *** et al.*** ** (2006) [** 16 **]**	MTA	80	6	10	10

***Laser: ***Since the early 1960s, lasers have been introduced to medicine and dentistry. Different lasers are used in pediatric dentistry. These lasers include diagnosis of caries development (diode 655 mm), argon lasers for composite curing, Co_2_ lasers with wavelength of 10600 nm for soft tissue surgeries, Nd: YAG lasers with wavelength of 1064 nm as well as diode laser with wavelength of 810-980 nm for soft tissue cutting, the Erbium laser family including Er: YAG (2940 nm) and Er; Cr: YSGG (2780 nm) which were used in hard tissues, cavity preparation and in soft tissue surgery and also low power lasers which are used in stimulatory and inhibitory biologic process [56]. Several studies have revealed that laser have proper effects in pulpotomy of primary teeth with results similar or even better than FS [20-22]. The advantages of laser compared to conventional pulpotomy, such as hemostasis, preservation of vital tissues near the tooth apex, absence of vibration and odor may lead to satisfaction of children and their parents. Nd: YAG laser with output power of 2 W and frequency of 20 Hz, Er:YAG laser with power of 0.5 W and frequency of 20 Hz, Co_2_ laser and 632/980 nm diode lasers can be used for pulpotomy of primary teeth [56-58].

Liu *et al.* [20] in a clinical study compared the effects of Nd: YAG laser pulpotomy with FC on human primary teeth. They concluded that the success rates of the Nd: YAG laser was significantly higher than the FC pulpotomy and the permanent successor of laser-treated teeth erupted without any complication.

Odabas *et al.* [22] reported that the clinical and radiographic success rates of Nd: YAG laser were 85.71 and 71.42%, respectively which were lower than success rates of FC pulpotomy. But there were no significant differences between laser and FC pulpotomy.

Based on a systematic review by De Coster *et al.* [57], laser has less success than conventional pulpotomy techniques and general recommendation for the clinical use of laser pulpotomy in primary teeth cannot be performed yet.

***Preservation***

In preservation methods, the pulp tissue is only minimally insulted. Preservation of pulpal tissue is exemplified by FS and NaOCl pulpotomy, which enable retention of maximum vital tissue and conservation of the radicular pulp without induction of reparative dentine.

***Ferric Sulfate: ***FS is a coagulative and hemostatic agent which is used for pulpotomies of primary teeth. Clinical and radiographical success rates for FS pulpotomies which were reported in several studies were 88-100% and 74-97%, respectively [26, 27, 29, 59-63]. A higher percentage of internal resorption is the major failure of FS pulpotomies reported by Papagiannoulis [63]. A recent systematic review and meta-analysis concluded that pulpotomies performed with either FC or FS in primary molars have similar clinical and radiographic success [64]. Furthermore, FS is inexpensive solution and no concerns about toxicity and carcinogenicity of FS have been recorded in dental literature [27]. Therefore, FS may be recommended as a suitable substitute for FC [64]. Table 3 represents the results of different studies regarding FS and FC pulpotomy. 

***Sodium Hypochlorite: ***NaOCl is the most widely used irrigating solution in endodontics due to its antimicrobial activity, tissue-dissolving property, detergent action, homeostasis and the ability to neutralize toxic products. Clinical and radiographic success rate of NaOCl pulpotomy were reported to be 100 and 76% respectively [36]. However, only few clinical trials evaluated the efficacy of NaOCl as a medicament in pulpotomy of primary teeth. A pilot study that investigated the use of 5% NaOCl by Vargas *et al.* [35] showed promising results after a 12-month period and a retrospective study conducted by Vostatek *et al*. [65] showed similar results. 

**Table 3:** Clinical and radiographic success rates of the studies comparing ferric sulfate (FS) and formocresol (FC) pulpotomy

**Author, year **	**Clinical success N (%)**		**Radiographic success N (%)**		**Follow-up (Month)**	**Sample size (n)**	
	**FC**	**FS**	**FC**	**FS**		**Baseline**	**Final**
**Fei ** ***et al.*** ** (1991) [** 59 **]**	26 (96.3)	29 (100)	22 (81)	28 (97)	12	FC=27	27
						FS=29	29
**Fuks ** ***et al. *** **(** **1997) ** **[** 60 **]**	31 (83.8)	51 (92.7)	27 (73)	41 (74.5)	35	FC=37	37
						FS=55	55
**Papagiannoulis (2002) [** 63 **]**	58 (97.3)	66 (90.3)	47 (78.3)	54 (74)	36	FC=60	60
						FS=73	73
**Ibrevic and Al-Jame (2003) [** 26 **]**	78 (97.5)	81 (96.4)	75 (91.7)	77 (93.7)	42-48	FC=80	80
						FS=84	84
**Huth ** ***et al.*** ** (2005) [** 61 **]**	44 (96)	42 (100)	43 (93.4)	42 (100)	24	FC=50	46
						FS=50	42
**Markovic ** ***et al. *** **(2005) [** 29 **]**	30 (90.9)	33 (89.2)	28 (84.8)	30 (81.1)	18	FC=34	34
						FS=37	37
**Farrokh Gisoure (2011) [** 27 **]**	24 (100)	27 (96.4)	21 (87.5)	24 (85.7)	9	FC=24	24
						FS=28	28
**Havale ** ***et al. *** **(2013) [** 50 **]**	23 (76.7)	29 (96.7)	17 (56.7)	19 (63.3)	12	FC=30	30
						FS=30	30

The results of a study by Al-Mutairi and Bawazir [66], showed that the clinical and radiographic success rate of 5% NaOCl pulpotomy were 94.6 and 86.5%, respectively after 12 months which was comparable to that of FC pulpotomy.

Ruby *et al*. [67] compared the clinical and radiographic success of vital pulpotomy treatment in primary molars using 3% NaOCl versus a 1.5 dilute of Buckley’s FC. They reported that NaOCl showed 100% clinical success and 90% radiographic success and there were no significant differences between NaOCl and FC success rates. This finding is in accordance with Shabzendedar *et al. *[68] who reported no significant difference between these materials. 

According to the studies mentioned above, it can be concluded that the clinical and radiographic success rates for NaOCl are comparable to FC pulpotomy in primary teeth but more randomized clinical trials must be conducted to conclude reliable decisions.

***Regeneration***

Formation of reparative dentine and preservation of healthy pulp tissue is rationale of regeneration approach which is done by several biomaterials and medicaments mentioned below: 

***Calcium hydroxide: ***CH was introduced to dentistry in 1838 by Nygren [69]. In 1930, Hermann showed that CH stimulated the formation of new dentin when placed in contact with human pulp tissue. Regarding VPT, CH was used as medicament for IDPC, DPC and pulpotomy in permanent and primary teeth, because of its bactericidal effect and ability to stimulate dentin bridge formation [70]. However, there are controversies regarding the use of CH in primary teeth pulpotomy, because it results in the development of chronic pulpal inflammation and internal resorption [71]. 

**Table 4:** Clinical and radiographic success rates of the studies comparing MTA and formocresol (FC) pulpotomy

**Author, year**	**Clinical success N (%)**		**Radiographic success N (%)**			**Follow-up (Month)**	**Sample size (n)**	
	**FC**	**MTA**	**FC**	**MTA**			**Baseline**	**Final**
**Agamy ** ***et al.*** ** (2004) [** 72 **]**	18 (90)	19 (100)	18 (90)		19 (100)	12	GMTA=20 FC=20	19 20
**Jabbarifar** *** et al.*** ** (2004) [** 73 **]**	29 (91)	30 (94)	29 (91)		30 (94)	12	MTA=64 FC=64	63 63
**Farsi ** ***et al.*** ** (2005) [** 74 **]**	35 (97.2)	38 (100)	31 (86.8)		38 (100)	24	MTA=60 FC=60	38 36
**Holan** *** et al.*** ** (2005) [** 48 **]**	24 (83)	32 (97)	24 (83)		32 (97)	4-74	MTA=33 FC=29	33 29
**Saltzman ** ***et al.*** ** (2005) [** 21 **]**	(13)100	(7)100	11 (84.6)		5 (71.4)	15.7±3	MTA=52 FC=52	20
**Naik and Hedge (2005) [** 75 **]**	23 (100)	24 (100)	23 (100)		24 (100)	6	MTA=25 FC=25	24 23
**Aeinehchi** *** et al.*** ** (2007) [** 76 **]**	57 (100)	43 (100)	47 (90.5)		43 (100)	6	MTA=43 FC=57	43 57
**Subramaniam ** ***et al.*** ** (2009) [** 15 **]**	20 (100)	20 (100)	17 (85)		19 (95)	24	MTA=20 FC=20	- -
**Zealand** *** et al.*** ** (2010) [** 77 **]**	100 (97)	100 (100)	89 (86)		95 (95)	6	GMTA=100 FC=103	100 103
**Ansari and Ranjpour (2010) [** 24 **]**	14 (93.3)	15 (100)	13 (90)		14 (95)	24	MTA=20 FC=20	15 15
**Hugar and Deshpande (2010) [** 78 **]**	30 (100)	30 (100)	29 (96.67)		30 (100)	36	MTA=30 FC=30	30 30
**Erdem** *** et al.*** ** (2011) [** 79 **]**	18 (72)	24 (96)	18 (72)		24 (96)	24	MTA=25 FC=25	25 25
**Godhi** *** et al.*** ** (2011) [** 80 **]**	25 (100)	25 (100)	22 (88)		24 (96)	12	MTA=25 FC=25	25 25
**Srinivasan and Jayanthi [** 81 **]**	42 (91.3)	47 (100)	36 (78.26)		45 (95.74)	12	MTA=50 FC=50	47 46
**Sushynski ** ***et al.*** ** (2012) [** 82 **]**	65 (98)	65 (100)	50 (76)		62 (95)	24	GMTA=126 FC=126	65 66
**Airen ** ***et al.*** ** (2012) [** 83 **]**	30 (85)	34 (97)	19 (54.3)		31 (88.6)	24	MTA=35 FC=35	- -
**Mettlach** *** et al.*** ** (2013) [** 84 **]**	131 (99)	119 (100)	105 (79)		113 (95)	42	MTA=135	119

Huth *et al*. [58] compared the success rates of pulpotomy with Er: YAG laser, CH, FS and dilute FC in primary teeth. They concluded that after 36 months of follow-up, FS revealed the best treatment outcome among the used techniques, while CH resulted in the lowest success rates. However, no significant differences were detected between FC and any other techniques.

Markovic *et al.* [29] found no statistical differences in overall, clinical and radiographic success rates for CH, FC and FS pulpotomies in their 18-month follow-up study. However, CH had the lowest overall success rate among the medicaments.

Although the cause of the inflammation inducing internal resorption is not fully understood, some researchers believed that the formation of blood clot following pulpotomy procedure interferes with wound healing and induces chronic inflammation of the residual pulp [70]. Whereas others have asserted that internal resorption leading to pulpal inflammation before pulpotomy is an important factor in the failure of CH pulpotomies [85]. The clinical success rates of CH pulpotomy of primary teeth have ranged between 31 to 100 % [71].

***Mineral trioxide aggregate: ***As a member of hydraulic calcium silicate cements [31] MTA was introduced by Lee *et al.* [86] and patented in 1995 by Torabinejad and White [87]. MTA consists of tricalcium silicate, bismuth oxide, tetra calcium alumina-ferrite and calcium-sulphate dehydrate. When MTA is mixed with water, a colloid gel with a pH of 12.5 similar to that of CH is formed [24]. When MTA was first commercialized, it had a gray coloration but in 2002 a new formula was created, the white MTA, to improve on the tooth discoloration property exhibited by gray MTA.

The major benefits of MTA are biocompatibility, being bactericidal and induction of cementogenesis. Furthermore, sealing ability, dentinogenesis and osteogenesis make it the preferred choice for numerous clinical treatments such as DPC, apexogenesis and apexification in immature teeth [31, 88]. In primary teeth, MTA is predominantly used for DPC [11, 16] and pulpotomy procedures [48, 89, 90]. The overall success rates for MTA as a pulpotomy medicament in primary teeth range from 94 to 100 % [31] based upon meta-analysis [91], systematic reviews [30] and evidence base assessments [92]. It seems that the efficacy of MTA is superior to FC which is the gold standard in pulpotomy of deciduous teeth [72, 92]. A huge number of investigations about evaluation of clinical and radiographic success rates of MTA as a pulpotomy medicament in primary teeth were performed. In Table 4, the recent studies comparing MTA and FC are mentioned.

Shirvani *et al. *[93] also compared the treatment outcomes of MTA and CH in a systematic review/meta-analysis and revealed that for pulpotomy of vital primary molars, MTA has better treatment outcomes compared to CH.

***Calcium-enriched mixture (CEM) cement: ***CEM cement was introduced as an endodontic filling material. The major components of the cement powder are calcium oxide (CaO), sulfur trioxide (SO_3_), phosphorus pentoxide (P_2_O_3_) and silicon dioxide (SiO_2_). The physical properties of this biomaterial, such as flow, film thickness, primary setting time and setting in aqueous environments are favorable [94].

CEM cement has proper biocompatibility, it can induce hard tissue and hydroxyapatite formation and it can resist microbial re-entrance and has remarkable antibacterial activity [14].

Recently, a 2-year randomized clinical trial study on the treatment outcomes of MTA and CEM pulpotomy in primary molars was done by Malekafzali *et al.* [95]. Overall, clinical and radiographic success rates in both MTA/CEM groups were comparable without any significant differences after 36 months of follow-up. Therefore, it seem that CEM may be an effective pulp dressing biomaterial [96, 97] but further investigations require to confirm the effectiveness of CEM cement for pulpotomy of primary teeth.

Other materials have been also evaluated for pulp capping such as BioAggregate, Endosequence Root Repair Material (ERRM), Biodentin, and Theracal [98-100]. However, in order to reach a definite conclusion about these materials, further clinical investigations in primary teeth are needed.

## Discussion

Primary dentition is essential for arch length maintenance, mastication, speech and esthetic in children and preservation of primary teeth in an intact condition until eruption of permanent successors is critical. Pulp injuries due to caries and trauma may threat pulp vitality, so appropriate treatment such as IDPC, DPC and pulpotomy must be considered.

In deciduous teeth, the failure rate of DPC is high and according to guidelines of AAPD, DPC is not recommended for primary teeth. However, several studies present high success rate of DPC treatment with some biomaterials [13, 14].

Based on clinical success rates of IPT, which are more than 90% [5, 6, 8]; this procedure is recommended as a preferable method for treating primary teeth with deep caries and reversible pulp inflammation. Among different medicaments used for IDPC, RMGI presents higher success rate than the others [5, 6, 8]. IDPC is less expensive, has fewer potential side effects and does not exhibit early tooth exfoliation [7, 101].

Pulpotomy is still the most common treatment method in case of pulp exposure in symptom-free primary molars, but in most cases the success of pulpotomy decreases overtime from ≥90% during the first 6-12 months to ≤70% after 36 months or more [8]. However, among different techniques and medicaments used in primary molar pulpotomy, the MTA pulpotomy appears to have a higher long-term success rate (>90%) [48, 64, 73]. On the other hand almost all of the studies on MTA pulpotomy have rather small sample size (*n*<50) [81] and have been done in short duration (<36 months) [78] and thus they may not be reliable enough to draw strong conclusions.

Primary molar pulpotomy has some side effects. Internal root resorption is one the most unfavorable outcomes stemming from chronic inflammation of residual radicular pulp [102]. This may be attributed to diagnostic errors made during assessing pulp condition or to technical failure while performing the selective procedure. Furthermore, early exfoliation of pulpotomized teeth is another side effect. More than 35% of FC pulpotomized teeth exfoliate earlier (≥6 months) than non pulpotomized teeth [8, 101].

Another complication is dentigerous cyst forming in permanent successors of pulpotomized deciduous teeth which were reported in several studies [103-105]. So, accurate diagnosis of pulp status and proper techniques are essential for success of pulpotomy and if some doubts about condition of pulp exist, the other methods such as pulpectomy or extraction must be considered.

## Conclusion

We can conclude in this literature review that:

1. IDPC is a favorable technique for treating primary teeth with deep caries without exposure of the reversibly inflamed pulp; it offers the advantages of lower cost, long-term higher success rate, and better exfoliation pattern.
2. DPC has not been recommended for primary teeth until now. Some new biomaterials present desirable result but long-term evaluation must be considered.

MTA pulpotomy is the most successful procedure among various types of pulpotomy in primary molar, but further randomized clinical trials with large sample size and long-term follow-up must be conducted.
